# Effect of lower esophageal gastric tube implantation in postoperative enteral nutritional support in patients with laryngeal cancer: A study protocol for a randomized controlled trial

**DOI:** 10.1097/MD.0000000000019771

**Published:** 2020-04-17

**Authors:** Hongying Xiao, Jianmin Liu, Sisi Liu, Xiaofang Chen

**Affiliations:** Department of Otolaryngology, Head and Neck Surgery, People's Hospital of De Yang City, Deyang City, Sichuan, China.

**Keywords:** cancer prognosis, comfort, gastric tube implantation, laryngeal cancer, postoperative care

## Abstract

**Background:**

For a long time, postoperative nutritional support for laryngeal cancer patients has depended on the gastric tube for enteral nutrition. Silica gel gastric tube is often used in clinical practice; however, the gastric tube placed in the conventional depth often leads to various complications in the stomach, thus damaging the nutritional status of patients and leading to the poor prognosis.

**Methods/design:**

A total of 80 patients with laryngeal cancer in otolaryngology, head and neck surgery department of Deyang people's hospital from May 2020 to April 2022 will be selected and randomly divided into control group and experimental group according to the numerical table. Patients in the control group will receive conventional gastric tube placement, with a depth of 45 to 55 cm, which can extract gastric juice. B-ultrasound accurately positioned the gastric tube in the stomach instead of the cardia, and postoperative nasal feeding nutrition will be provided. In the experimental group, the gastric tube will be pulled out 10 cm after conventional placement and no gastric juice will be extracted. B-ultrasonography verified that the gastric tube will be located below the esophagus or above the cardia, and routine nasal feeding will be performed postoperatively. Analysis for comfort and prognosis were performed by general comfort questionnaire and various index including height, body mass index, albumin value, electrolyte, wound healing, pharyngeal fistula.

**Discussion:**

In this study, visual simulation scale and general comfort questionnaire developed by Kolaba, an American comfort nursing specialist, were used to evaluate the comfort level of the 2 groups of patients, including pain, acid reflux, upper abdominal burning sensation, and hiccup. Objective indexes such as height, body mass index, albumin value, electrolyte, wound healing, and pharyngeal fistula were used to evaluate the prognosis of the 2 groups of patients. The visual simulation scale can preliminarily judge the subjective feelings of patients.

**Trial registration:**

It has been registered at http://www.chictr.org.cn/listbycreater.aspx (Identifier: ChiCTR2000030378), Registered February 29, 2020.

## 1 Introduction

Laryngeal cancer is a common malignant tumor in otolaryngology head and neck surgery. All patients undergoing laryngeal cancer surgery need tracheotomy; however, the postoperative recovery process is slow. Indwelling of nasal feeding tube and tracheostomy have a great impact on the quality of life of patients.^[1]^ In terms of clinical status, more than 500 cases of laryngeal cancer operations were performed in our department from January 2012 to December 2020, and most of the patients complained of chest and anterior cardiac discomfort, acid reflux, burp, and gas discomfort, which brought huge psychological pressure and physical pain to patients. Comfort care is a holistic, individualized, creative and effective nursing mode, which can make people achieve pleasure in both physiology and psychology, thus reducing the society burden.^[2]^ The change of comfort level of patients with nasal feeding is the main reason for patients’ self-extubation.^[3]^ Therefore, it is particularly urgent to conduct clinical research on the selection of reasonable gastric tube placement depth for patients with laryngeal cancer. According to the clinical practice in our department, we found that most patients have significantly reduced discomfort after 10 cm of gastric tube is removed. To verify the rationality of this method, we plan to conduct a randomized controlled trial protocol in the present study.

## 2 Methods/design

### 2.1 Research object

We aim to explore the optimal depth of gastric tube placement in perioperative period for laryngeal cancer patients.

We aim to compare and analyze the differences in postoperative comfort and prognosis between the traditional gastric tube placement method (control group) and the modified lower esophageal gastric tube placement method (experimental group).

### 2.2 Study method

A total of 80 patients with laryngeal cancer in otolaryngology, head and neck surgery department of Deyang people's hospital from May 2020 to April 2022 will be selected and randomly divided into control group and experimental group according to the numerical table. Analysis for comfort and prognosis were performed by general comfort questionnaire (GCQ)^[4]^ and various index including height, body mass index, albumin value, electrolyte, wound healing, pharyngeal fistula. By statistical analysis, the effects of 2 different gastric tube placement depths on comfort and nutritional status of laryngeal cancer patients will be evaluated.

### 2.3 Participants

Inclusion criteria include the following:

(1) Eighty cases of laryngeal cancer surgery patients in otolaryngology head and neck surgery from March 2020 to September 2020 will enroll in this study.
(2) Patients who deny a previous history of digestive disease will enroll in this study.
(3) Patients who have normal cognitive, behavioral and communication skills will enroll in this study.
(4) Patients who volunteer to participate in this study and sign the informed consent will enroll in this study.

Exclusion criteria include the following:

(1) Patients with hypopharyngeal cancer who have undergone chemoradiotherapy will be excluded;
(2) Laryngeal cancer patients with serious internal and surgical diseases, such as hyperthyroidism, hypertension, heart disease, hepatitis will be excluded;
(3) Illiteracy will be excluded.

### 2.4 Interventions

Patients in the control group will receive conventional gastric tube placement, with a depth of 45 to 55 cm, which can extract gastric juice. B-ultrasound accurately positioned the gastric tube in the stomach instead of the cardia, and postoperative nasal feeding nutrition will be provided. In the experimental group, the gastric tube will be pulled out 10 cm after conventional placement and no gastric juice will be extracted. B-ultrasonography verified that the gastric tube will be located below the esophagus or above the cardia, and routine nasal feeding will be performed postoperatively.

### 2.5 Measurement of outcomes

Tools to measure primary indicators include the following:

(1) The incidence of gastric complications.
(2) In this study, visual simulation scale and GCQ developed by Kolaba,^[4]^ an American comfort nursing specialist, will be used to evaluate the comfort level of the 2 groups of patients, including pain, acid reflux, upper abdominal burning sensation, and hiccup.
(3) Objective indexes such as height, body mass index, albumin value, electrolyte, wound healing, and pharyngeal fistula will be used to evaluate the prognosis of the 2 groups of patients.

### 2.6 Statistical analysis

Statistical analyses will be implemented by SPSS 17.0 and Microsoft Excel 2007 software. Data will be represented as mean ± standard deviation. A *t* test will be performed to compare the changes in measures within groups. Statistical significance will be considered at *P* < .05.

## 3 Discussion

Laryngeal cancer is a common malignant tumor in otolaryngology, head and neck surgery in China.^[5]^ Throat excision is the main treatment for laryngeal cancer patients. After surgery, patients need the indwelling gastric tube nasogastric feeding to obtain the nutrition required^[6]^; however, gastric tube indwelling for a long time can cause a series of complications, such as gastric retention, diarrhea, hiccups, nasogastric tube, aspiration, and pharyngeal fistula.^[7]^ In addition, indwelling nasogastric feeding tube can also make the patient appear acid reflux, sternal pain, stomach burning sensation, and other discomfort, resulting in decreased appetite of the patient.^[8]^ Feeding tube nutrition after laryngeal cancer plays an important role in the recovery of surgery and the improvement of patients’ quality of life, and the nursing of nasal feeding tube is an important part of perioperative nursing of laryngeal cancer patients.^[9,10]^ The conventional preoperative or intraoperative gastric tube placement depth is 40 to 55 cm, but the gastric tube placement depth makes most patients complain of chest and precardiac discomfort, such as stomach distension, cauterization, acid reflux, hiccup, and so on, which seriously affects the patient's tolerance and recovery.

The visual simulation scale can preliminarily judge the subjective feelings of patients. GCQ contains a total of 30 questions enrolling in 4 dimensions with 5 physical questions, 10 mental questions, 7 environmental questions, 8 social cultural questions; in addition, a total of 10 positive questions and 20 negative questions. Therefore, the simplified GCQ-Chinese translation has a high reliability and validity, which can reflect the comfort status of patients well and is applicable to all kinds of people.

This protocol aims at revealing the influence of 2 different gastric tube placement methods on postoperative comfort and prognosis of laryngeal cancer patients. This protocol may verify the rationality of an improved gastric tube placement method and has far-reaching clinical application significance for improving postoperative life quality and comfort of laryngeal cancer patients.

## Author contributions

Hongying Xiao conceived the idea for this study; Xiaofang Chen provided statistical plan; Sisi Liu drafted the protocol. Jianmin Liu reviewed the protocol and provided critical feedback. All authors approved the article in its final form.
